# Facial Rejuvenation With an Innovative Poly‐l‐Lactic Acid (Juläine) for Nasolabial Folds: Interim Data Analysis of a Prospective, Non‐Randomized, Multicenter, Open‐Label Spanish Study

**DOI:** 10.1111/jocd.70137

**Published:** 2025-03-26

**Authors:** Fernando Urdiales‐Gálvez, Paula A. Benítez, Iratxe Díaz

**Affiliations:** ^1^ Instituto Médico Miramar Málaga Spain; ^2^ Clínica ROBEGA Madrid Spain; ^3^ Clínica Iratxe Díaz Bilbao Spain

To the Editor,

Collagen, comprising 80% of human skin, depletes with age, accelerating skin aging by relaxing collagen and elastin fibers, which lead to wrinkles and surface depressions [1].

Poly‐l‐lactic acid (PLLA) fillers, widely used in Europe and the Americas, provide a safe and effective alternative for facial wrinkles, with results lasting up to one year [2, 3, 4].

This article presents preliminary results from a prospective and multicenter study conducted across three Spanish centers, evaluating the efficacy and safety of the new PLLA‐LaSynPro (Juläine, Nordberg Medical AB, Sweden), a next‐generation injectable PLLA designed to stimulate collagen production. The trial focused on facial volume deficits and skin laxity in the nasolabial folds. This interim analysis primarily assessed safety outcomes while evaluating the effectiveness of the PLLA‐LaSynPro filler.

The study enrolled 36 patients, all aged 18 years or older, with mild to severe nasolabial folds as determined by a Wrinkle Severity Rating Scale (WSRS) [5] score of ≥ 2 on both sides of the face (Supporting Information).

The primary endpoint of this interim analysis was the incidence of adverse events. Secondary endpoints included the proportion of subjects achieving at least a 1‐point reduction in the WSRS [5], a reduction of ≥ 1 point in the middle third facial volume loss scale (MDFDS), the proportion of patients rated as “much better” or “much improved” on the Global Aesthetic Improvement Scale (GAIS), and procollagen type I carboxy‐terminal propeptide (P1CP) levels measured 2 months after the first treatment dose (Figure 1).

**FIGURE 1:**
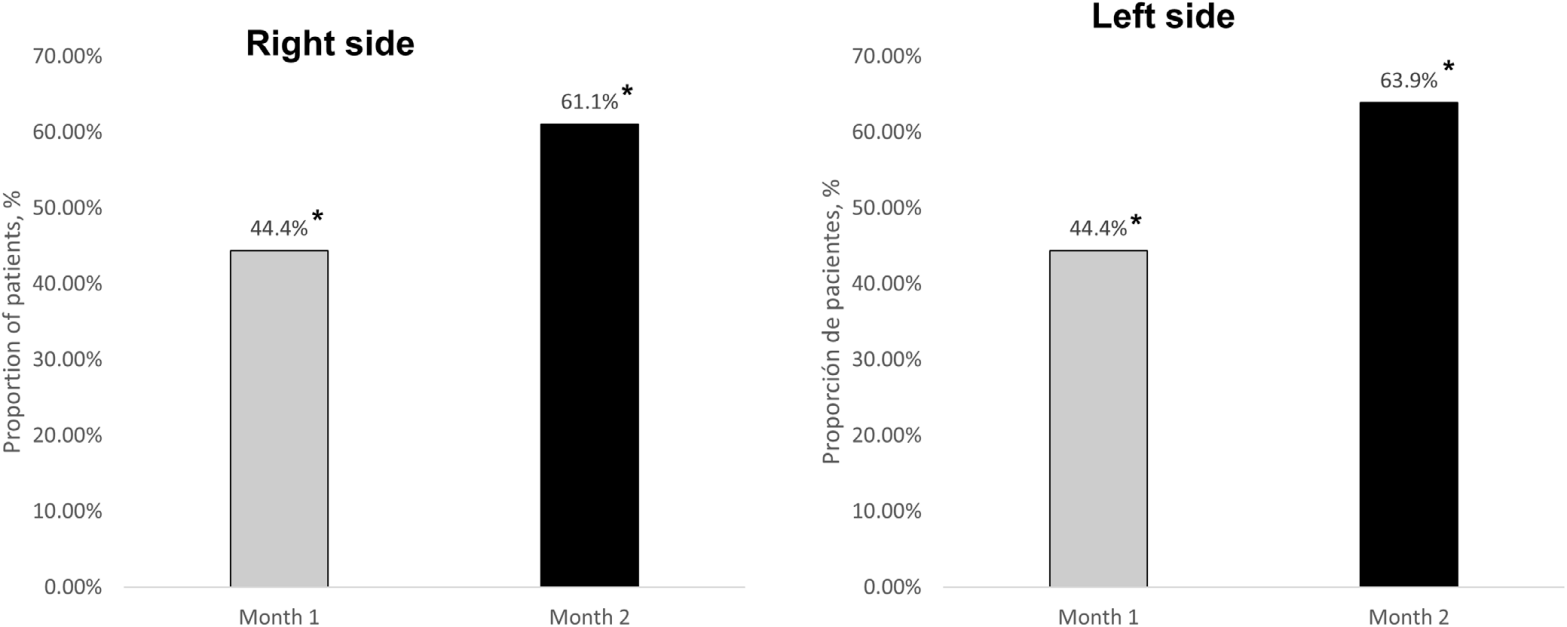
Proportion of patients achieving a ≥ 1 point reduction in Wrinkle Severity Rating Scale (WSRS) nasolabial fold depth 2 months following the first dose of poly‐l‐lactic acid (PLLA) LaSynPro filler treatment. A 1‐point reduction in the WSRS was observed in 16.7% of patients between month 1 and month 2 of treatment (*p* = 0.1587). *p < 0.0001, compared to baseline.

The chi‐squared test will be used to assess changes in qualitative variables throughout the study. Changes in blood levels of P1CP will be analyzed using Friedman's two‐way analysis of variance, with pairwise comparisons performed using the Conover post hoc method. A *p*‐value of < 0.05 will be considered statistically significant.

The study results showed that most adverse events (AEs) were mild and transient. Mild inflammation was the most common, affecting 61.1% and 55.6% of subjects 1 week after the first and second treatment doses, with rare moderate cases (2.8%). Edema and erythema were also mild, with minimal occurrences and no severe cases. Overall, AEs were infrequent, mild in intensity, and transient, with a low incidence of moderate or severe events (Table 1).

**TABLE 1:** Incidence of adverse events (AEs).

	V0	V1	V2^a^	V3	V4^b^	V5
Inflammation, *n* (%)						
Mild	7 (19.4)	22 (61.1)	18 (33.3)	12 (33.3)	20 (55.6)	14 (38.9)
Moderate	0 (0.0)	0 (0.0)	0 (0.0)	2 (6.5)	0 (0.0)	0 (0.0)
Severe	0 (0.0)	1 (2.8)	0 (0.0)	1 (2.8)	1 (2.8)	0 (0.0)
Edema, *n* (%)						
Mild	1 (2.8)	0 (0.0)	0 (0.0)	0 (0.0)	0 (0.0)	0 (0.0)
Moderate	2 (5.6)	0 (0.0)	2 (5.6)	0 (0.0)	2 (5.6)	0 (0.0)
Severe	0 (0.0)	0 (0.0)	0 (0.0)	0 (0.0)	0 (0.0)	0 (0.0)
Erythema, *n* (%)						
Mild	5 (13.9)	0 (0.0)	6 (16.7)	0 (0.0)	4 (11.2)	0 (0.0)
Moderate	1 (2.8)	0 (0.0)	1 (2.8)	0 (0.0)	1 (2.8)	0 (0.0)
Severe	0 (0.0)	0 (0.0)	0 (0.0)	0 (0.0)	0 (0.0)	0 (0.0)

*Note:* During the study follow‐up period, two patients reported experiencing “mild discomfort at the injection site” and the presence of “a small bump” in the injection area. V0, before the first treatment; V1, 1 week ±1 day after the first treatment; V2, before the second treatment; V3, 1 week ±1 day after the second treatment; V4, before the third treatment; V5, 1 week ±1 day after the third treatment.

^a^ 1 month ±1 week after the first treatment.

^b^ 1 month ±1 week after the second treatment.

On the WSRS scale, 44.4% of patients achieved a ≥ 1‐point reduction in wrinkle severity 1 month after the first dose, increasing to 63.9% at 2 months (*p* < 0.0001 each, as compared to baseline). The improvement was uniform on both the right and left sides.

On the MFVDS, 44.4% of patients showed a ≥ 1‐point reduction in mid‐face volume loss 1 month after the first dose, increasing to 63.9% at 2 months (*p* < 0.0001 each, as compared to baseline). Improvements were balanced on both the right and left sides (Figure S2).

Patient satisfaction also demonstrated positive outcomes, with 66.7% of patients reporting they felt “Much Better” or “Better” 1 month after the first dose. Following the second dose, 63.9% expressed the same level of satisfaction 2 months post‐treatment, relative to their condition at month one (Figure S3).

P1CP levels significantly increased 1 month after the first dose (*p* = 0.0040) and remained stable, with no notable changes between Month 1 and Month 2 (Figure S4).

This new PLLA‐LaSynPro filler contains fully absorbable polylactide microspheres.

Preliminary results from this study indicated that PLLA‐LaSynPro filler had a favorable safety profile when used under appropriate conditions. Additionally, it effectively restored volume, corrected skin laxity, improved facial contours, and enhanced overall skin quality.

Further research is required to assess long‐term outcomes and optimize treatment protocols to enhance patient satisfaction and results durability. Additionally, evaluating mid‐ and long‐term safety outcomes is crucial for identifying the ideal patient profile, ensuring maximum therapeutic benefit while minimizing potential risks.

## Author Contributions

F.U‐G. designed and directed the project, the main conceptual ideas, and proof outline. P.A.B. and I.D. drafted the manuscript, conducted the literature search, and designed the tables and figures. F.U‐G., P.A.B., and I.D. collected data. Critical review and edition of the manuscript were performed. All authors reviewed the results and approved the final version of the manuscript.

## Conflicts of Interest

Dr. Urdiales‐Gálvez received research grants to cover the costs of medical writing services and publication fees, honoraria for lectures, and travel support to attend educational meetings from Nordberg. Dr. Benítez has no financial interests to declare. Dr. Díaz has no financial interests to declare.

## Supporting information


Figure S1.

Figure S2.

Figure S3.

Figure S4.

Data S1.

Table S1.

## Data Availability

The data that support the findings of this study are available on request from the corresponding author. The data are not publicly available due to privacy or ethical restrictions.
