# Supplementary figures and images for: Facial Rejuvenation With an Innovative Poly‐l‐Lactic Acid (Juläine) for Nasolabial Folds: Interim Data Analysis of a Prospective, Non‐Randomized, Multicenter, Open‐Label Spanish Study
Source: J Cosmet Dermatol. 2025 Mar 26;24(4):e70137. doi: 10.1111/jocd.70137 (PMC11938402; doi:10.1111/jocd.70137)

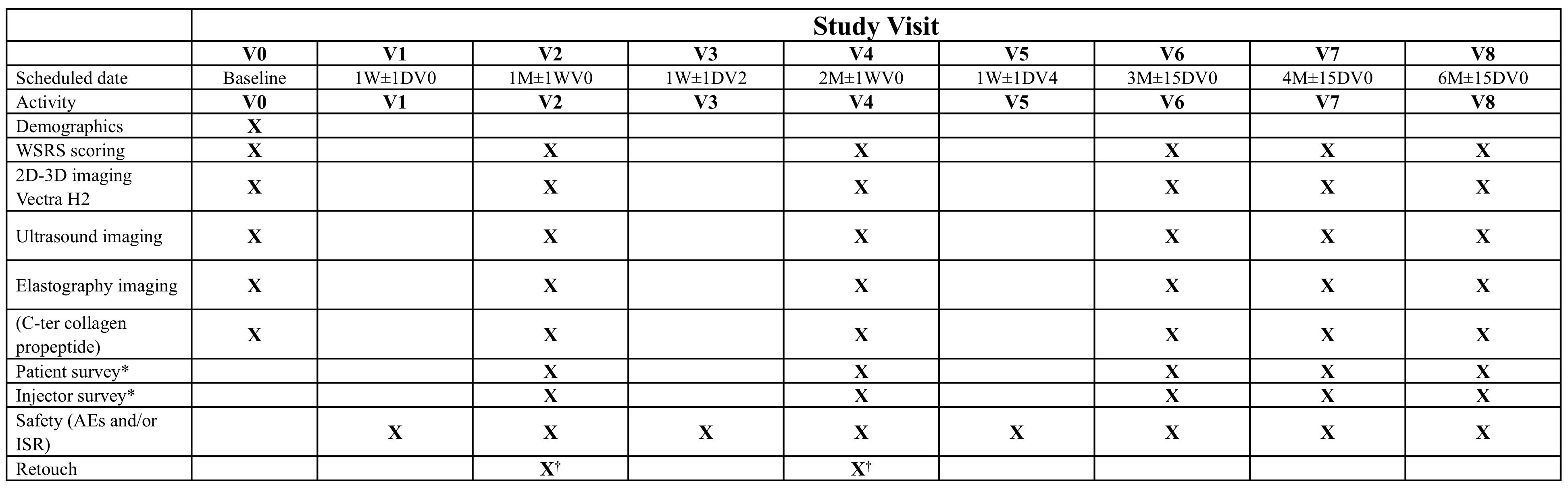

Supplement: Supplementary file 1 — Figure S1. [file JOCD-24-e70137-s003.TIF]

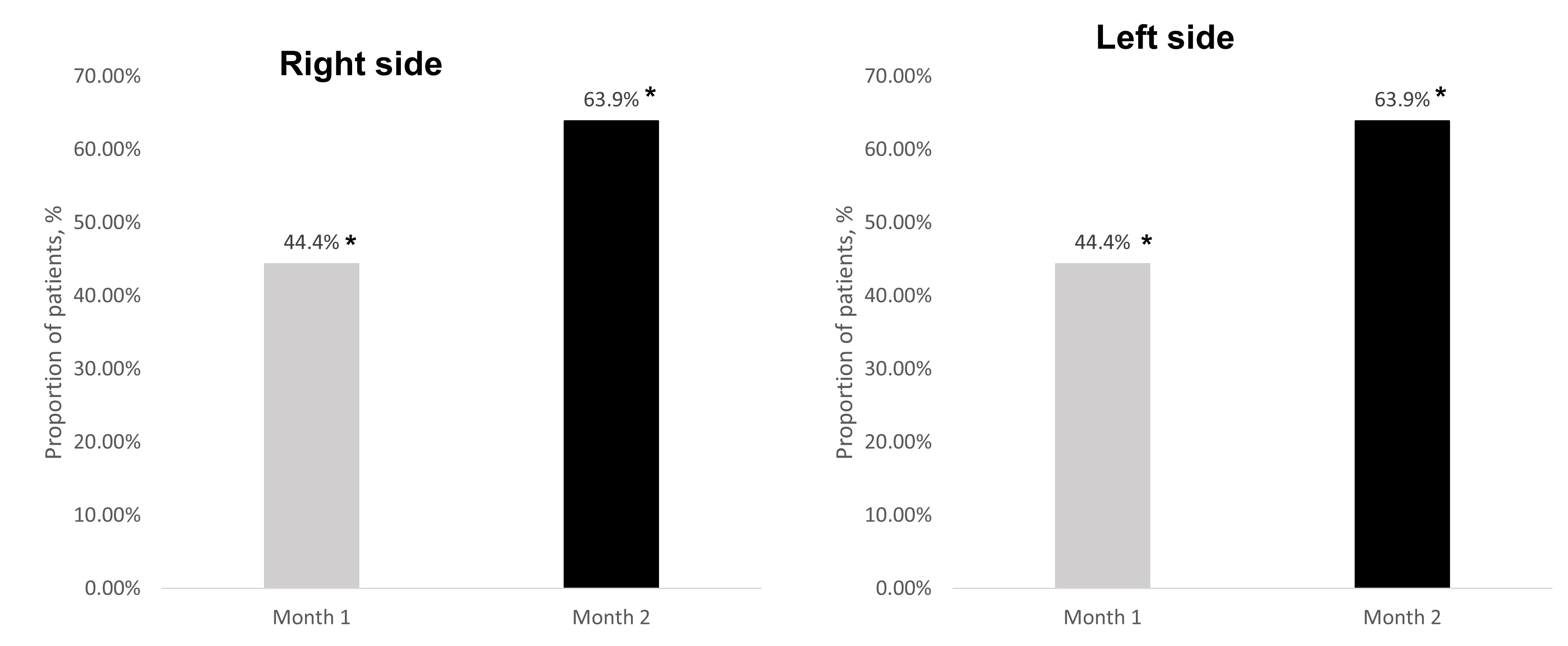

Supplement: Supplementary file 2 — Figure S2. [file JOCD-24-e70137-s004.TIF]

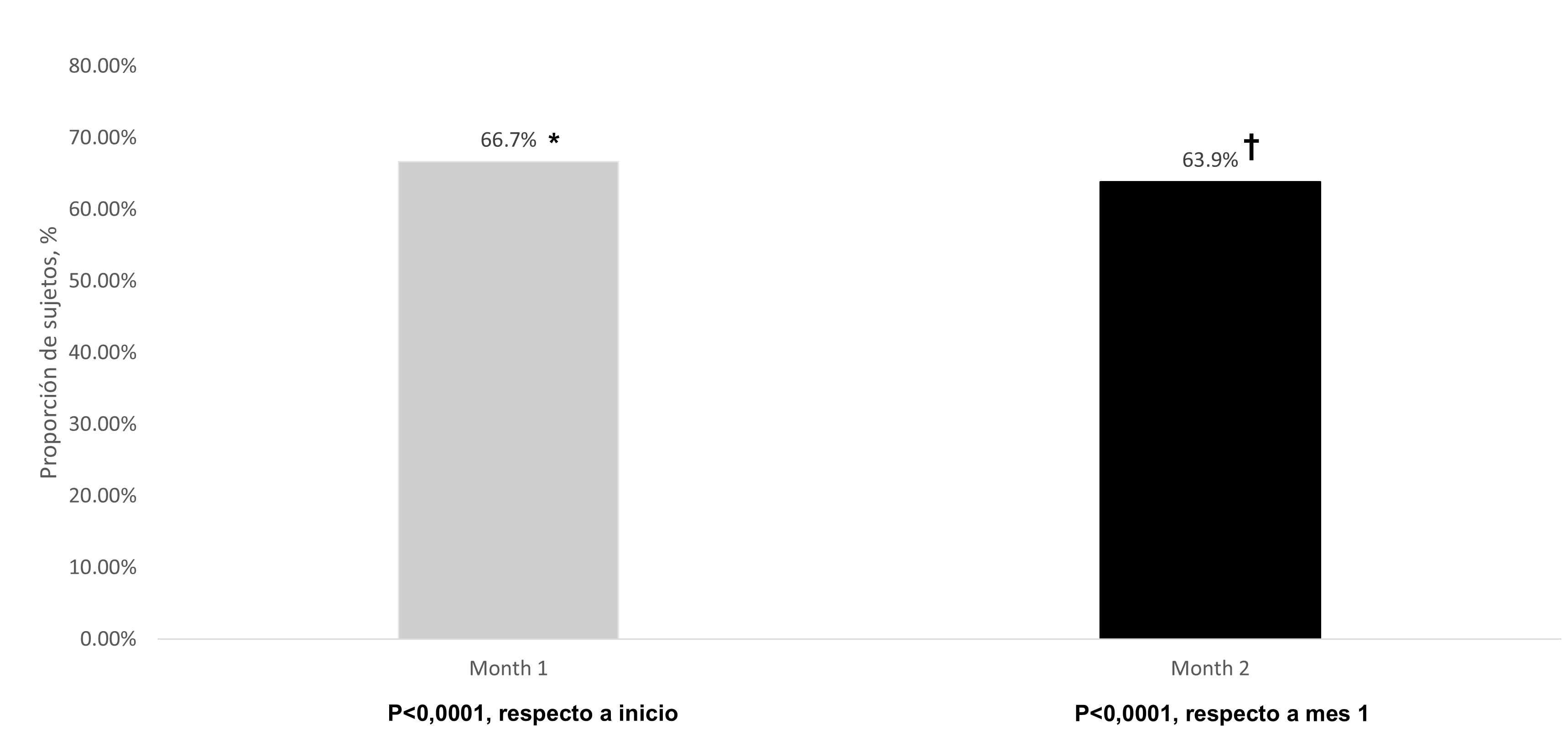

Supplement: Supplementary file 3 — Figure S3. [file JOCD-24-e70137-s002.TIF]

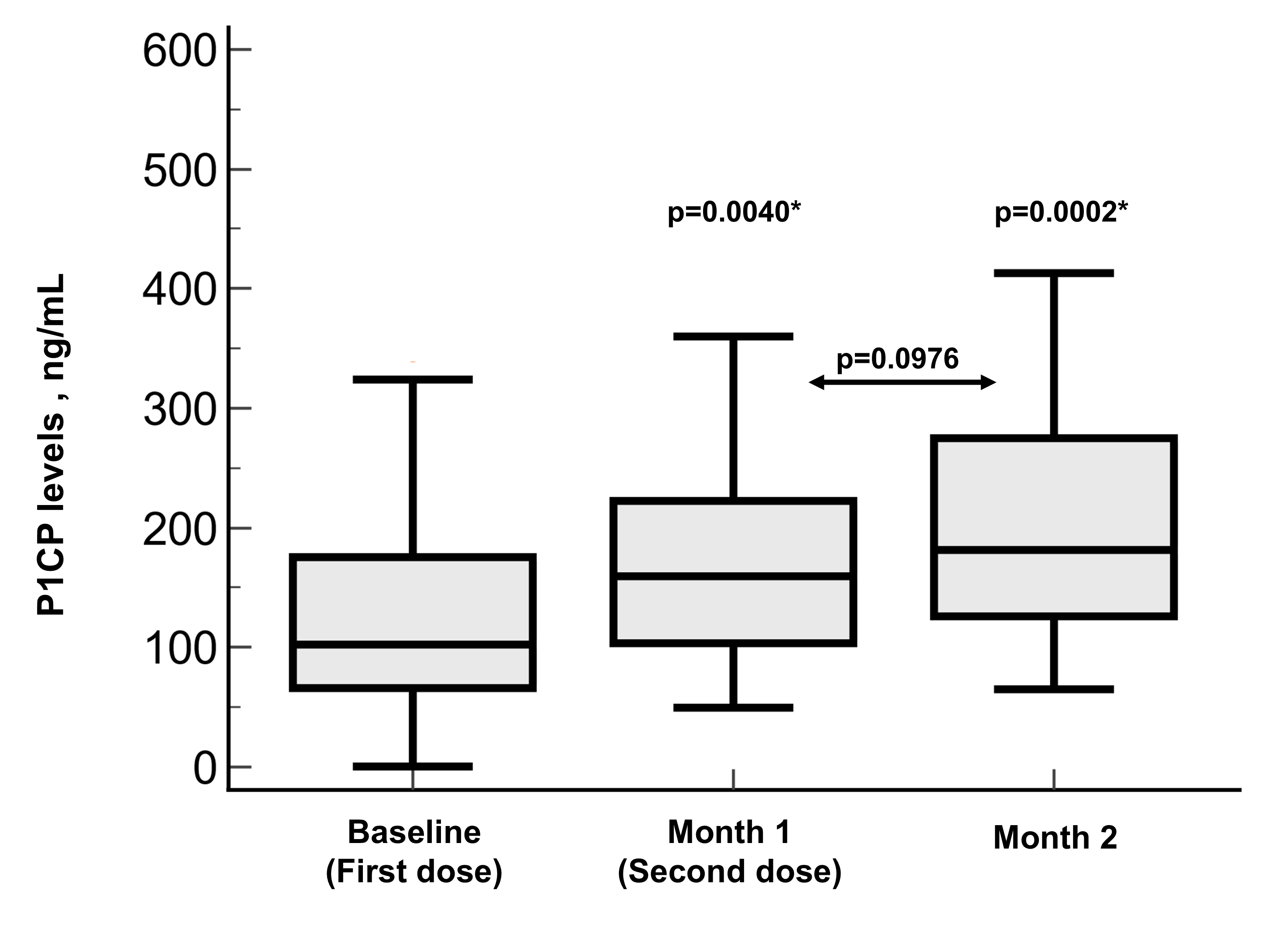

Supplement: Supplementary file 4 — Figure S4. [file JOCD-24-e70137-s006.TIF]
