# Supplementary material for: Facial Rejuvenation With an Innovative Poly‐l‐Lactic Acid (Juläine) for Nasolabial Folds: Interim Data Analysis of a Prospective, Non‐Randomized, Multicenter, Open‐Label Spanish Study
Source: J Cosmet Dermatol. 2025 Mar 26;24(4):e70137. doi: 10.1111/jocd.70137 (PMC11938402; doi:10.1111/jocd.70137)
Supplement: Supplementary file 5 — Data S1. [file JOCD-24-e70137-s001.docx]

**Facial Rejuvenation with an innovative Poly-l-lactic Acid (Juläine) for Nasolabial Folds: Interim data Analysis of a Prospective, Non-Randomized, Multicenter, Open-Label Spanish Study.**

**Supplementary Material**

**Methods**

*Study Objective*

To assess the effectiveness and safety of the Polylactic acid (PLLA)-LaSynPro^TM^ filler in subjects with facial volume deficit and skin laxity in the nasolabial fold.

*Study design*

This study was a prospective, multicenter, open-label, non-randomized, and non-controlled trial using a single-group design. All participants will receive the new PLLA-LaSynPro^TM^ (Juläine^TM^, Nordberg Medical AB, Sweden). Data analysis will be conducted in a masked manner, ensuring the analyst remains unaware of the study product.

The study adhered to Good Clinical Practice (GCP) guidelines, the Declaration of Helsinki, and applicable country-specific regulations, prioritizing whichever offered greater individual protection. Written informed consent was obtained from all participants prior to enrollment. To ensure anonymity, any identifying information was encrypted or removed as necessary. The study protocol was reviewed and approved by the Ethics Committee of the Instituto Médico Miramar.

*Study Participants*

Subjects, either women or men, with facial volume deficit and skin laxity in the nasolabial fold.

To be eligible for the study, participants must be ≥18 years old, immune-competent, able to provide written informed consent, and have mild to severe nasolabial folds assessed by the Wrinkle Severity Rating Scale (WSRS) (1) (score ≥2 on both sides of the face). They must also be willing to comply with the study protocol and refrain from other facial procedures during the study. Exclusion criteria include prior facial surgery or dermal filler treatments for nasolabial fold correction, recent local treatments on the face below the zygomatic arch, pigmentation or hypo melanosis in the nasolabial folds, keloid or hypertrophic scarring, known allergies to specific substances (e.g., lidocaine, PLLA), history of herpes eruptions, malignant skin disorders, or other serious diseases, known hemorrhagic disease, use of anti-coagulants, active or recurrent infections, recent use of immunosuppressants or steroids, conditions affecting wound healing, pregnancy or nursing, and women planning to become pregnant during the study. Additionally, participants with any other conditions that may interfere with study procedures may also be excluded.

*Treatment Protocol*
Participants will receive treatment according to the approved labeling, which involves up to three injections of the product into the deep dermis or subcutaneous layer of the nasolabial folds. Injections will occur at baseline, week 4, and week 8, with each visit having a window of ±1 week. Follow-up visits will be scheduled one week ±1 day after each injection for safety monitoring, and at 1, 3, and 6 months after the final PLLA-LaSynPro^TM^ injection to assess both efficacy and safety. The total duration of patient participation in this phase of the study will not exceed 10 months (Figure S1).

*Determining the Degree of Severity of Wrinkles*

The Wrinkle Severity Rating Scale

The WSRS is a clinically validated tool designed to objectively assess the severity of facial wrinkles. This scale employs a 5-point ordinal system, where each point corresponds to a specific degree of wrinkle severity (1):

1. **Absent (0):** No visible wrinkles.
2. **Mild (1):** Shallow but visible wrinkles.
3. **Moderate (2):** Moderately deep wrinkles.
4. **Severe (3):** Deep wrinkles with well-defined edges.
5. **Extreme (4):** Very deep and long wrinkles, creating prominent folds.

The midface volume deficit scale

The Midface Volume Deficit Scale (MFVDS) is a six-point photonumeric scale developed by Allergan® as a standardized physician-assessment tool for evaluating the overall severity of midface volume loss (2,3). This scale categorizes volume deficits into six distinct grades, ranging from 0 (none) to 5 (severe), enabling objective and reproducible clinical assessments. The specific grading classifications are as follows: none [0], minimal [1], mild [2], moderate [3], significant [4], and severe [5] (2,3). A detailed description of each grade within the MFVDS is provided in Table S1.

*Study Outcomes*

The primary end-point od this interim analysis was the incidence of adverse events.

The secondary end-points were proportion of subject who achieved at least 1 point reduction in WSRS; reduction ≥ 1 point in the middle third facial volume loss scale (MDFDS); proportion of patients rated as "much better" / "much improved" on the Global Aesthetic Improvement Scale (GAIS), and procollagen type I carboxy-terminal propeptide (P1CP) levels 2 months after the first treatment dose.

*Statistical analysis*

Sample Size

The sample size calculation assumed that 75% of participants would achieve a minimum 1-point improvement in the WSRS score at month 6 compared to baseline. To account for a type I error (α) of 0.05 and a type II error (β) of 0.2, approximately 29 subjects are required. A statistical power of 80% was selected to minimize the risk of a false negative result. To account for a potential 20% patient withdrawal rate, a minimum of 36 patients will be recruited.

Statistical analysis was conducted using MedCalc® Statistical Software version 23.0.6 (MedCalc Software Ltd, Ostend, Belgium; [https://www.medcalc.org](https://www.medcalc.org/); 2024).

Data has been presented as median values with 95% confidence intervals (95% CI) or interquartile range (IQR), mean ± standard deviation (SD), or percentages, depending on the data type. Changes in blood levels of P1CP was analyzed using Friedman’s two-way analysis of variance. Pairwise comparisons were conducted using post hoc analysis with the Conover method. The Chi-squared test was employed to evaluate changes in WSRS scores over the course of the study. A p-value of less than 0.05 will be considered statistically significant.

**Results**

On the WSRS scale, 44.4% of patients achieved a ≥1-point reduction in wrinkle severity one month after the first dose (p<0.0001), increasing to 63.9% at two months (p<0.0001). The improvement was consistent on both the right and left sides (Figure S2).

On the MFVDS, 44.4% of patients achieved a ≥1-point reduction in mid-face volume loss one month after the first dose (p<0.0001 vs. baseline), increasing to 63.9% at two months (p<0.0001 vs. baseline). Reductions were consistent and significant on both the right and left sides (Figure S3).

Patient satisfaction mirrored these improvements, with 66.7% reporting feeling "Much Better" or "Better" one month after the first dose (p<0.0001 vs. baseline). After the second dose, 63.9% reported similar satisfaction compared to their status at month one (p<0.0001 vs. baseline) (Figure S3).

P1CP levels significantly increased from baseline (133.4 ± 100.3 ng/mL) to one month (182.4 ± 96.5 ng/mL; p=0.0040) and two months (210.9 ± 117.7 ng/mL; p=0.0040) after the first PLLA dose. However, no significant difference was observed between Month 1 and Month 2 (mean difference: 28.6 ± 100.8 ng/mL; 95% CI: -5.5 to 62.7 ng/mL; p=0.0976) (Figure S4)

**References**

1. Day DJ, Littler CM, Swift RW, Gottlieb S. The wrinkle severity rating scale: a validation study. Am J Clin Dermatol. 2004;5(1):49-52.

2. Jones D, Murphy DK. Volumizing hyaluronic acid filler for midface volume deficit: 2-year results from a pivotal single-blind randomized controlled study. Dermatol Surg. 2013;39(11):1602-12.

3. Urdiales-Gálvez F, Delgado NE, Figueiredo V, Lajo-Plaza JV, Mira M, Ortíz-Martí F, et al. Preventing the Complications Associated with the Use of Dermal Fillers in Facial Aesthetic Procedures: An Expert Group Consensus Report. Aesthetic Plast Surg. 2017;41(3):667-677.

**Figure Legends**

Figure S1. Study follow-up time-table.

*Degree of satisfaction with the of poly-L-lactic acid (PLLA) LaSynPro^TM^ filler treatment.

^†^If needed.

W: Week; D: Day, M: Month; WSRS: Wrinkle Severity Rating Scale; AEs: Adverse Events; ISR: Injection Site Reactions.

Figure S2. Proportion of patients achieving a ≥1 point reduction in middle third facial volume loss scale (MDFDS) 2 months following the first dose of poly-L-lactic acid (PLLA) LaSynPro^TM^ filler treatment.

A 1-point reduction in the MDFDS was observed in 19.5% of patients between month 1 and month 2 of treatment (p = 0.0992).

*p<0.0001, compared to baseline

Figure S3. Proportion of patients who are Much better/better compared to baseline (Month 1) and compared to Month 1 (Month 2) visit in patient satisfaction rating according to Global Aesthetic Improvement Scale (GAIS).

Sixty-six-point seven percent of patients reported feeling "Much Better" or "Better" one month after the first dose of poly-L-lactic acid (PLLA) LaSynPro^TM^ filler treatment, compared to their baseline condition. Additionally, 63.9% of patients reported the same improvement one month after the second dose (two months following the first poly-L-lactic acid (PLLA) LaSynPro^TM^ filler treatment dose), compared to their condition at Month 1.

*p<0.0001, compared to baseline

†p<0.0001, compared to Month-1 visit.

Figure S4. Procollagen type I carboxy-terminal propeptide (P1CP) levels throughout the study follow-up.

Collagen C-terminal pro-peptide levels significantly increased from baseline (133.4 ± 100.3 ng/mL) to one month (182.4 ± 96.5 ng/mL; p=0.0040) and two months (210.9 ± 117.7 ng/mL; p=0.0040) after the first PLLA dose. However, no significant difference was observed between Month 1 and Month 2 (mean difference: 28.6 ± 100.8 ng/mL; 95% CI: -5.5 to 62.7 ng/mL; p=0.0976).
