# Supplementary material for: Facial Rejuvenation With an Innovative Poly‐l‐Lactic Acid (Juläine) for Nasolabial Folds: Interim Data Analysis of a Prospective, Non‐Randomized, Multicenter, Open‐Label Spanish Study
Source: J Cosmet Dermatol. 2025 Mar 26;24(4):e70137. doi: 10.1111/jocd.70137 (PMC11938402; doi:10.1111/jocd.70137)
Supplement: Supplementary file 6 — Table S1. [file JOCD-24-e70137-s005.docx]

Table S1. Allergan® midface volume deficit scale. Adapted from Jones & Murphy (2) and Urdiales-Gálvez et al (3).

| **Grade** | **Definition** |
| --- | --- |
| **0 None** | - Moon face - *Fullness* (*convexity*) in the zygomaticomalar region, anteromedial cheek, and/or submalar region |
| **1 Minimal** | - *Flattening* in the zygomaticomalar region, anteromedial cheek, and/or submalar region |
| **2 Mild** | - *Mild concavity* in the zygomaticomalar region, anteromedial cheek, and/or submalar region - Mild *tear troughs and/or nasolabial folds* |
| **3 Moderate** | - *Moderate concavity* in the zygomaticomalar region, anteromedial cheek, and/or submalar region - Moderate tear troughs and/or nasolabial folds - Mild *nasojugal folds and/or prejowl sulcus* - Mild prominence of bony landmarks - Mild visibility of musculature |
| **4 Significant** | - *Significant concavity* in the zygomaticomalar region, anteromedial cheek, and/or submalar region - Significant tear troughs and/or nasolabial folds - Moderate nasojugal folds and/or prejowl sulcus - Moderate prominence of bony landmarks - Moderate visibility of musculature |
| **5 Severe** | - *Wasting* - Severe concavity in the zygomaticomalar region, anteromedial cheek, and/or submalar region - Severe tear troughs and/or nasolabial folds - Significant nasojugal folds and/or prejowl sulcus - Significant prominence of bony landmarks - Significant visibility of underlying musculature |
